# A study of capecitabine metronomic chemotherapy is non-inferior to conventional chemotherapy as maintenance strategy in responders after induction therapy in metastatic colorectal cancer

**DOI:** 10.1186/s13063-020-4194-6

**Published:** 2020-03-06

**Authors:** Min Shi, Tao Ma, Wenqi Xi, Jinling Jiang, Junwei Wu, Chenfei Zhou, Chen Yang, Zhenggang Zhu, Jun Zhang

**Affiliations:** 1 Department of Oncology, Ruijin Hospital, Shanghai Jiaotong University School of Medicine, No. 197 Ruijin Er Road, Shanghai, 200025 China; 2 Department of Surgery, Shanghai Institute of Digestive Surgery, Ruijin Hospital, Shanghai Jiaotong University School of Medicine, No. 197 Ruijin Er Road, Shanghai, 200025 China

**Keywords:** Metronomic chemotherapy, Capecitabine, Maintenance treatment, Metastatic colorectal cancer

## Abstract

**Background:**

The aim of this study is to demonstrate that capecitabine metronomic chemotherapy is non-inferior to capecitabine conventional chemotherapy as maintenance treatment, in patients who have responded to 16–18 weeks first-line chemotherapy in metastatic colorectal cancer (mCRC).

**Methods:**

The study design is a prospective, randomized, open label, phase II clinical trial. Those patients with mCRC who respond well after 16–18 weeks of standard doublet chemotherapy as induction may be enrolled into this study, and randomly assigned to the capecitabine metronomic group or standard dosage group. The duration of disease control after randomization and progression-free survival after enrollment are the primary endpoints. Overall survival, safety, and quality of life are the secondary endpoints. The sample size required to achieve the research objectives of this project is 79 patients in each group. The study recently started on 1 January 2018, and will last for 36 months.

**Discussion:**

This project is intended to study the efficacy and safety of capecitabine metronomic chemotherapy in the maintenance treatment of advanced colorectal cancer, and to explore the strategy of “low toxicity, high efficiency, economy, and individualization”, which is suitable for China’s national conditions and pharmacoeconomics. It has great prospects for clinical application and a clear socioeconomic value.

**Trial registration:**

ClinicalTrials.gov: NCT03158610. Registered on 15 May 2017.

## Background

Global cancer statistics for 2018 [1] indicated that there would be an estimated 18.1 million newly diagnosed cancer cases and 9.6 million cancer-related deaths in 2018. Among them, over 1.8 million new colorectal cancer cases and 881,000 deaths were estimated to occur in 2018. Overall, colorectal cancer ranked third in all cancer incidence (6.1%) and second for mortality (9.2%) [1]. The incidence rates of colorectal cancer are about threefold higher in transitioned versus transitioning countries [1]. The difference may due to dietary patterns, obesity, and lifestyle factors. Standard screening and early detection programs have been conducted in the USA and Japan since the 1990s [2], and the 5-year survival rate in colorectal cancer increased from 51% (1990) to 65% (2012), while more and more patients were diagnosed with early-stage cancer [3]. Even so, almost half of patients with colorectal cancer will eventually develop metastasis and lose the chance to eradicate cancer [4]. How to prolong survival in these patients and inhibit the growth of tumors on the premise of guaranteeing the quality of life, and manage metastatic colorectal cancer (mCRC) as a chronic disease like diabetes mellitus or hypertension through long-term, low-toxicity, and effective drug treatment is of great clinical research value.

Drugs for treatment of mCRC have ranged from 5-fluorouracil monotherapy in the 1960s to 5-fluorouracil in combination with oxaliplatin or irinotecan and with or without targeted agents such as bevacizumab, cetuximab, or panitumumab in the past decade. The median overall survival (OS) of patients with mCRC was from less than 12 months to more than 33 months [5–11]. However, conventional chemotherapy usually gives the maximum tolerable dose of the drug, and will cause huge toxicity and side effects while killing cancer cells. Chemotherapy-related vomiting, diarrhea, agranulocytosis, peripheral neurotoxicity and other serious adverse reactions occur as in 5–20% of patients [7, 12–14]. It takes a period of time for the body to recover from toxic effects and side effects after each routine chemotherapy administration, and repeated multiple cycles of administration are more likely to cause toxicity accumulation, which limits the number of courses of treatment. More importantly, after a period of high-intensity chemotherapy, how to continue to effectively and persistently inhibit the progress of cancer, while ensuring patients have good tolerance and quality of life, has been a hot topic in cancer research, but also a clinical problem to be solved urgently.

Metronomic chemotherapy is a low-dose, high-frequency mode of continuous administration of antineoplastic drugs without long intermission [15]. The recommended dose is only 1/10–1/3 of the maximum tolerable dose of the drug, so the incidence and intensity of treatment-related side effects are greatly reduced. The antineoplastic mechanism of metronomic chemotherapy is not directed against cancer cells and therefore it will not produce the problem of drug resistance induced by small doses of drugs. By inhibiting the proliferation and migration of vascular endothelial cells, metronomic chemotherapy is also known as “anti-angiogenesis chemotherapy” [16].

## Methods

### Aim of the study

The aim of this study is to demonstrate that capecitabine metronomic chemotherapy is non-inferior to capecitabine conventional chemotherapy as maintenance treatment in patients with mCRC who have responded to 16–18 weeks of first-line chemotherapy.

### Study design

**Fig. 1:**
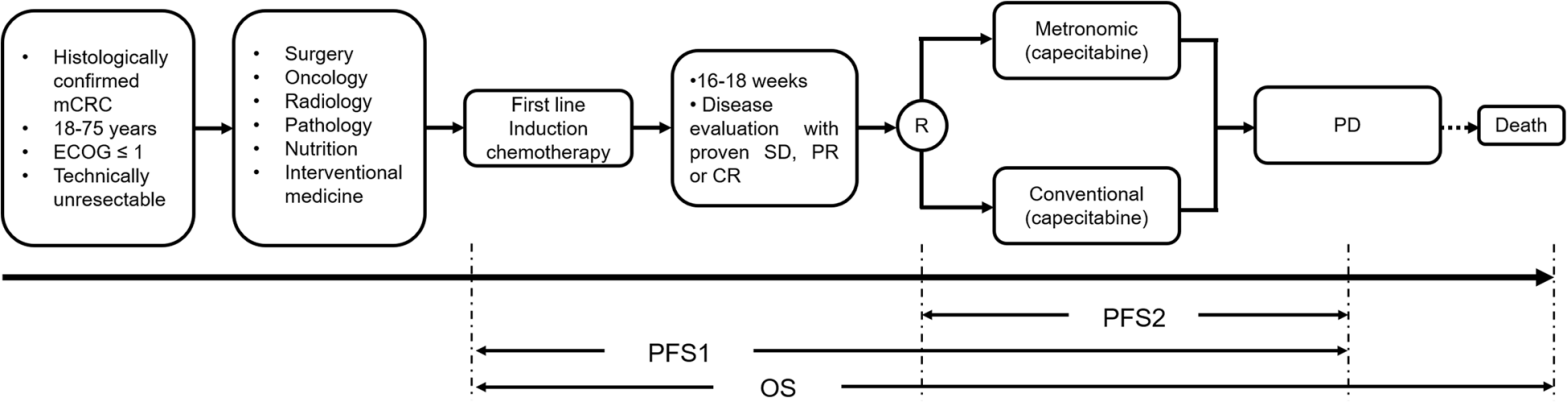
Study design and flowchart. mCRC, metastatic colorectal cancer; ECOG, Eastern Cooperative Oncology Group; SD, stable disease; PR, partial response; CR, complete response; PD, progression of disease; PFS, progression-free survival; OS, overall survival

**Fig. 2:**
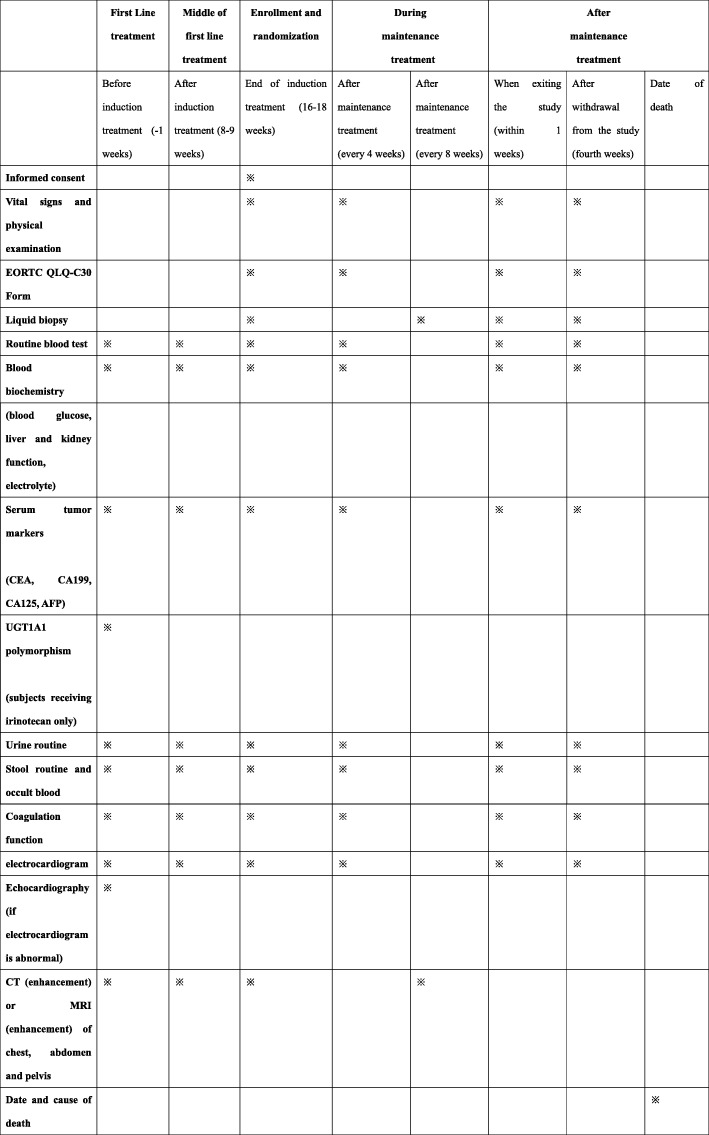
Standard protocol items: recommendation for interventional trials (SPIRIT) figure. EORTC QLQ-C30, European Organization for Research and Treatment of Cancer Quality of Life Questionnaire; CT, computed tomography; MRI, magnetic resonance imaging

The study design is a prospective, randomized, open label, phase II clinical trial (Fig. 1). Patients with mCRC who respond well, have stable disease (SD), and partial response (PR) or complete response (CR) according to the response evaluation in solid tumors (RECIST) criteria after 16–18 weeks of standard doublet chemotherapy as induction, may be enrolled into this study, and randomly assigned to the capecitabine metronomic group or standard dosage group. Patients are randomized by a sealed envelope system. The maintenance treatments are continued until disease progression or severe toxicity. Furthermore, exploratory markers involving angiogenesis (serum vascular endothelial growth factor (VEGF), platelet-derived growth factor (PDGF), Tie-1 and Tie2, etc.) and immune function (CD clusters, serum tumor mutation burden (TMB), etc.), are measured via liquid biopsy (Fig. 2).

### Study objectives

The duration of disease control after randomization (progression-free survival 2, PFS2) is the primary endpoint. Progression-free survival from induction treatment (PFS1), overall survival (OS), safety, and quality of life (QoL) are the secondary endpoints.

### Study population

The study population consists of patients with unresectable metastatic colorectal cancer, who are scheduled for treatment with first-line doublet chemotherapy. Patients’ inclusion and exclusion criteria are defined as follows.

Inclusion criteria are:

1. Patients of age 18–75 years;
2. Histopathologically confirmed colorectal adenocarcinoma classified as technically unresectable (patients with only local recurrence are not eligible);
3. No prior first-line treatment with chemotherapy, radiotherapy, immunotherapy, or targeted therapy; adjuvant chemotherapy is allowed if it has been more than 6 months since the treatment was finished and there have been no signs of disease progression, neither during treatment nor during the 6 months following its completion;
4. Life expectancy > 12 weeks;
5. Eastern Cooperative Oncology Group (ECOG) performance status ≤ 1;
6. At least one measurable lesion for assessment by computed tomography (CT) or magnetic resonance imaging (MRI);
7. Adequate bone marrow function (Hb > 6.0 mmol/L, absolute neutrophil count > 1.5 × 109/L, platelets > 100 × 109/L), renal function (serum creatinine ≤ 1.5 × upper limit of normal (ULN) and creatinine clearance, Cockroft formula, > 30 ml/min), liver function (serum bilirubin ≤ 2 × ULN, serum transaminases ≤ 3 × ULN without presence of liver metastases or ≤ 5 × ULN with presence of liver metastases);
8. Disease evaluation with proven SD, PR or CR according to RECIST criteria after first-line induction treatment before randomization;
9. Written informed consent obtained before randomization;

Exclusion criteria are:

1. Brain metastasis with large amounts of pleural and abdominal effusion;
2. Pregnancy or breastfeeding;
3. Disease evaluation with progression of disease (PD) according to RECIST criteria after first-line induction treatment;
4. Previous systemic treatment for advanced disease;
5. Major surgery or radiotherapy (except for antalgic surgery that does not include measurable target lesions) during the 4 weeks prior to inclusion in the study;
6. Participation in another clinical trial with use of drugs within the previous 30 days;
7. Neoplasm in the 2 years prior to entering the study, except for non-melanoma skin carcinoma or in situ cervix carcinoma;
8. Symptomatic heart disease (arrhythmia, heart failure, or history of myocardial infarction);
9. Active infection, active bleeding, or serious metabolic disorder;
10. Signs and symptoms of acute or subacute bowel obstruction at the time of entering the study;
11. Chronic immunological or hormonal treatment, except for hormone replacement treatment at physiological doses;
12. Any geographical or social circumstance or any medical or psychological alteration that, in the investigator’s opinion, will not allow the patient to conclude the study.

### Study protocol

#### MDT

Ideally, patients will be discussed by the multi-disciplinary team (MDT) for colorectal cancer from the Departments of Surgery, Oncology, Radiology, Pathology, Nutrition and Interventional medicine, etc.) of Ruijin Hospital, Shanghai Jiaotong University School of Medicine, with first-line chemotherapy regimen formulated by the joint consultation of these experts.

#### First-line treatment regimens

Standard doublet chemotherapy is used as induction treatment, which includes mFOLFOX6 regimen (oxaliplatin 85 mg/m2 intravenous (iv) d1, leucovorin 400 mg/m2 iv d1, 5-fluorouracil 400 mg/m2 iv d1, 5-fluorouracil 2400 mg/m2 continuous intravenous (CIV) 46 h, q2w), FOLFIRI regimen (irinotecan 180 mg/m2 iv d1, leucovorin 400 /m2 iv d1, 5-fluorouracil 400 mg/m2 iv d1, 5-fluorouracil 2400 mg/m2 CIV 44 h, twice weekly (q2w)), XELOX regimen (oxaliplatin 135 mg/m2 iv d1, capecitabine 1000 mg/m2 bid po d1–14, q3w), XELIRI regimen (irinotecan 250 mg/m2 iv d1, capecitabine 1000 mg/m2 bid po d1–14, q3w). The total number of first-line treatments is six cycles for XELOX/XELIRI regimens, and eight cycles for mFOLFOX6/FOLFIRI regimens.

#### Maintenance treatment regimens

Single-agent chemotherapy is used as maintenance treatment, which includes capecitabine metronomic chemotherapy (capecitabine 500 mg twice a day (bid) orally (po)) and capecitabine conventional chemotherapy (capecitabine 1000 mg/m2 bid po, d1–14, 3 times weekly (q3w).

#### Outcome measurements

Evaluation of tumor response is performed every 8 weeks using the RECIST criteria [17]. Toxicity is assessed after each cycle using National Cancer Institute Common Toxicity Criteria for Adverse Events (NCI-CTCAE) [18]. Quality of life is assessed after each cycle using the European Organization for Research and Treatment of Cancer Quality of Life Questionnaire.

### Sample size calculation

This project is a non-inferiority study. The patients are allocated to the capecitabine metronomic chemotherapy group (experimental group) and the capecitabine conventional chemotherapy group (control group) in a ratio of 1:1. The non-inferiority boundary in PFS was defined at 1.40 in reference to the results of the trial reported by Luo et al. [19]. The HR for the capecitabine maintenance group versus the observation group in the trial was 0.54 and the reciprocal was 1.85, which leads to 1.43 as 50% survival benefit. So, the boundary of 1.4 was used in this study. Considering a dropout rate of 20%, we estimated that 386 patients (193 in each group) would be needed to achieve 80% power at a one-sided value of α = 0.025 (significance level).

### Statistical analysis

Those patients who do not follow the protocol of their assigned treatment arm will not be analyzed. The statistical analysis will be carried out using SPSS software (version 17.0; SPSS, Chicago, IL, USA). Descriptive statistics will be used for safety evaluation. Mean values and standard deviations (SDs) will be provided for continuous endpoints and frequency and percentage distributions will be provided for discrete data. PFS and OS will be estimated using the Kaplan-Meier method and their medians with two-sided 95% CIs will be calculated. Comparisons between groups of patients will be made by the log-rank test. All statistical analysis will be carried out at a 5% level of significance.

## Discussion

Until recently, few patients with mCRC could tolerate full doses of chemotherapy longer than 4–6 months, with limitations that are mainly due to severe neurotoxicity (oxaliplatin) and chronic diarrhea (irinotecan) [20]. Thus, limiting the duration of the induction chemotherapy to a short period, then exploiting maintenance to prolong disease control at the price of a reasonable toxicity profile, is an appealing strategy for patients with mCRC [21].

Many studies have aimed to reduce the treatment burden and maintain a favorable outcome. The OPTIMOX1 trial compared 5FU/LV maintenance treatment with a continuous FOLFOX4 regimen in patients with mCRC and found that there were no significant difference in PFS, OS, or incidence of adverse events between the two groups, which suggests that fluorouracil could be used as an alternative maintenance therapy during the standard regimen treatment without affecting the overall therapeutic effect [22]. The MACRO trial compared the efficacy and safety of bevacizumab alone with bevacizumab plus capecitabine and oxaliplatin as maintenance treatment after induction chemotherapy in patients with mCRC, which suggests that single-agent bevacizumab as maintenance therapy may be an appropriate option following induction XELOX plus bevacizumab in patients with mCRC with mild improvement in PFS [23]. The MACRO 2 trial compared the efficacy and safety of cetuximab alone with cetuximab plus mFOLFOX as maintenance treatment after induction chemotherapy in patients with mCRC; there were no statistically significant differences in the PFS and OS, and the objective response rate and safety profile were also similar. This suggests that maintenance therapy with single-agent cetuximab following induction therapy with mFOLFOX plus cetuximab could be a valuable option compared with continued treatment with mFOLFOX plus cetuximab [24]. The CAIRO3 study was an evaluation of metronomic capecitabine combined with bevacizumab as maintenance treatment in patients with mCRC. The conclusion of the study was that PFS was significantly longer in the group receiving maintenance treatment with capecitabine metronomic chemotherapy and bevacizumab than it was in the observation group. Further, the incidence of chemotherapy-related leukopenia, peripheral neurotoxicity, and other serious toxic reactions was only increased by 5–10% in the maintenance group compared with the observation group, which was completely tolerated by the patients, and thus capecitabine metronomic chemotherapy combined with bevacizumab proved to be effective and low-toxicity maintenance therapy [25]. But bevacizumab is expensive; the cost-effectiveness of capecitabine and bevacizumab maintenance therapy for mCRC has been assessed, demonstrating that antineoplastic therapy is expensive for payers and society. The price of capecitabine and bevacizumab maintenance therapy needs to be reduced by 93% to make it cost-effective, which restricts the clinical application of this combination regimen as maintenance treatment in patients with mCRC.

Xu et al. [19] reported on the use of single-agent capecitabine as maintenance therapy after induction of first-line chemotherapy in mCRC: the primary endpoint of PFS in the capecitabine maintenance group (capecitabine 1000 mg/m2 bid po, d1–14, q3w) was 6.43 months (95% CI 5.26–7.71), which was significantly longer than in the observation group (3.43 months, 95% CI 2.83–4.16), HR = 0.54 (0.42–0.70), *P* < 0.001. However, the maximum tolerable dose of capecitabine for maintenance therapy was not adjusted, resulting in the incidence of grade 3/4 toxicity of up to 41% in the maintenance treatment group, in particular with significantly greater incidence of leukopenia, thrombocytopenia, hand-foot syndrome, and mucositis in the maintenance therapy group than in the observation group.

In recent years, metronomic chemotherapy, as a maintenance therapy strategy for advanced tumors, has increasingly been used in the clinic and has become a new hotspot of anti-cancer therapy. It is a promising strategy for inhibiting angiogenesis and is associated with lower toxicity than conventional chemotherapy [26]. Our previous publication reported that capecitabine metronomic chemotherapy decreased vascular endothelial growth factor (VEGF), while elevating expression of thrombospondin-1 (TSP-1), an endogenous inhibitor of angiogenesis. Capecitabine metronomic chemotherapy also reduced CEP levels and decreased microvessel density (MVD) [27]. Our findings indicated that targeting angiogenesis rather than drug-sensitive tumor cells explained the antitumor effects of capecitabine metronomic treatment in colon cancer cells. With regard to immunomodulation and tumor microenvironments, there are reports that metronomic chemotherapy could restore peripheral T-cell proliferation and activate the cytotoxicity of immune effector cells, which could inhibit tumor progression [28]. Besides this, metronomic chemotherapy could also upregulate dendritic cells to stimulate the proliferation of T lymphocytes [29]. Interestingly, reports suggest that metronomic chemotherapy could result in tumor immunogenicity with antigen processing and presentation genes.

The concepts were emphasized in the design of this study, which featured a shortened induction phase limited to 8 instead of 12 cycles (mFOLFOX6/FOLFIRI regimens) and 6 instead of 8 cycles (XELOX/XELIRI regimens), followed by two different maintenance strategies. Besides this, exploratory markers involving angiogenesis (serum VEGF, PDGF, Tie-1, and Tie2, etc.) and immune function (CD clusters, serum tumor mutation burden (TMB), etc.) are conducted via liquid biopsy.

In conclusion, this study is a prospective study evaluating whether the effect of capecitabine metronomic chemotherapy as maintenance treatment is non-inferior to capecitabine conventional chemotherapy, in patients with mCRC who have responded to 16–18 weeks first-line chemotherapy. This project is intended to study the efficacy and safety of capecitabine metronomic chemotherapy in the maintenance treatment of advanced colorectal cancer, and to explore the strategy of “low toxicity, high efficiency, economy and individualization”, which is suitable for China’s national conditions and pharmacoeconomics. It has great clinical application prospects and clear socioeconomic value.

## Trial status

This is protocol version 20,170,407. Enrollment started on 29 January 2018 and will last for 36 months. After the start of the study, the first 30 months will consist of inclusion and follow up of the patients. The last 6 months will consist of follow up and analysis of results. The study will end on 29 January 2021.

## Data Availability

All data generated or analyzed during this study are included in this paper.

## Abbreviations

CR: Complete response
CT: Computed tomography
ECOG: Eastern Cooperative Oncology Group
mCRC: Metastatic colorectal cancer
MDT: Multi-disciplinary team
MRI: Magnetic resonance imaging
MVD: Microvessel density
NCI-CTCAE: National Cancer Institute Common Toxicity Criteria for Adverse Events
OS: Overall survival
PD: Progression of disease
PDGF: Platelet-derived growth factor
PFS: Progression-free survival
PR: Partial response
QoL: Quality of life
RECIST: Response evaluation in solid tumors
SD: Stable disease
SDs: Standard deviations
TMB: Tumor mutation burden
TSP-1: Thrombospondin-1
ULN: Upper limit of normal
VEGF: Vascular endothelial growth factor
